# Independent evolution of highly variable, fragmented mitogenomes of parasitic lice

**DOI:** 10.1038/s42003-022-03625-0

**Published:** 2022-07-08

**Authors:** Andrew D. Sweet, Kevin P. Johnson, Stephen L. Cameron

**Affiliations:** 1 Department of Biological Sciences, Arkansas State University, Jonesboro, AR 72401 USA; 2 Illinois Natural History Survey, Prairie Research Institute, University of Illinois, Champaign, IL 61820 USA; 3 Department of Entomology, Purdue University, West Lafayette, IN 47907 USA

**Keywords:** Molecular evolution, Evolutionary genetics

## Abstract

The mitochondrial genomes (mitogenomes) of bilaterian animals are highly conserved structures that usually consist of a single circular chromosome. However, several species of parasitic lice (Insecta: Phthiraptera) possess fragmented mitogenomes, where the mitochondrial genes are present on separate, circular chromosomes. Nevertheless, the extent, causes, and consequences of this structural variation remain poorly understood. Here, we combined new and existing data to better understand the evolution of mitogenome fragmentation in major groups of parasitic lice. We found strong evidence that fragmented mitogenomes evolved many times within parasitic lice and that the level of fragmentation is highly variable, including examples of heteroplasmic arrangements. We also found a significant association between mitochondrial fragmentation and signatures of relaxed selection. Mitochondrial fragmentation was also associated with changes to a lower AT%, possibly due to differences in mutation biases. Together, our results provide a significant advance in understanding the process of mitogenome fragmentation and provide an important perspective on mitochondrial evolution in eukaryotes.

## Abstract (web summary)

Multiple independent origins of fragmented mitochondrial genome evolution in parasitic lice provide insights into eukaryotic mitochondrial evolution.

## Introduction

Most animals have mitochondrial genomes (mitogenomes) that consist of a single, circular chromosome that is between 12,000–18,000 base pairs (bp) long. This conserved structure is likely a result of the crucial and nearly universal mitochondrial function in eukaryotic cells, as deviations from this structure are usually associated with cell death and/or disease^1–4^. However, there are a few notable exceptions scattered across the animal kingdom. The most pronounced examples of variant mitogenome structure are in organisms outside of Bilateria, including in some hydrozoa and jellyfish^5–8^, which have mitogenomes that consist of multiple (in some cases hundreds), fragmented linear chromosomes. Within Bilateria, fragmented mitogenome chromosomes are only known from a genus of nematode (*Globodera*)^9^ and three groups of insects: thrips^10^, book lice^11^, and parasitic lice^12^. In all four of these cases, the mitogenomes consist of multiple smaller, circular chromosomes. Of these, parasitic lice have the most extreme variation in mitogenome structure^13,14^. However, the extent and causes of mitogenome fragmentation in lice remain largely unknown.

Fragmented mitogenomes in lice were first described in the human body louse (*Pediculus humanus corporis*), which have 20 circular fragments (“minicircles”) each containing 1–3 genes^12^. Subsequent studies indicated that fragmented mitogenomes in lice were ancestral in a clade of “mammal” lice (Parvorders Anoplura, Rhynchophthirina, and Trichodectera)^14–19^ including the human louse, but more recent studies suggest mitogenome fragmentation is more widespread, although perhaps sporadic, across lice^13^. In agreement with these findings, full or partial mitogenomes with multiple chromosomes have been reported from a genus in Ischnocera (*Columbicola*)^20^ and from several genera in Amblycera^21^, indicating that mitogenome fragmentation evolved multiple times within lice. However, despite these recent developments, it is not known how common fragmented mitogenomes are in parasitic lice, nor how frequently fragmentation has evolved in the group. In addition, levels of mitochondrial fragmentation and gene arrangements on the fragments can even be variable within a genus of louse^14,20^. However, it is unknown how stable the mitogenome organization is among individuals (or populations) within a species of louse. Heteroplasmy can also be present in some mitogenomes of lice, either in the form of divergence between homologous genes or multiple chromosomal arrangements within a single individual^13,18,22^. These reported cases suggest heteroplasmy could be much more prevalent in parasitic lice, particularly in lice with fragmented mitogenomes.

The causes and consequences of mitogenome fragmentation in lice are also mostly unknown, although several molecular mechanisms responsible for fragmentation have been proposed, including recombination in the mitogenomes^12^ and a lack of key nuclear genes involved in mitogenome replication^13^. There is also evidence that fragmented mitogenomes in lice have a much higher rate of substitution, likely due to underlying differences in mutation rates, compared to lice with single-chromosome mitogenomes^23^. In this case, we would expect a signature of relaxed selection associated with the evolution of mitogenome fragmentation.

In conjunction with high substitution rates and nonadaptive processes, fragmentation could also lead to a shift in nucleotide composition of the mitogenomes. Most insects (and animals more generally) have highly AT-biased (usually >70% AT) mitogenomes^24^. However, previous work has reported that lice with fragmented mitogenomes have a more balanced (less AT-biased) nucleotide composition, whereas lice with single mitogenomes have base compositions more similar to other insects^20,25^. This difference could be related to mutation biases. Specifically, a lower AT% in fragmented mitogenomes could be a result of fewer deamination mutations occurring during replication^26^, because the chromosome fragments are smaller and replication can occur in a shorter time period. However, these hypotheses have not been explored in detail beyond observations of general patterns.

Here, we assemble, annotate, and compare mitochondrial genomes across a broad diversity of lice to understand the extent, causes, and consequences of mitogenome fragmentation in parasitic lice. Specifically, we address the following questions: (1) how prevalent are fragmented mitogenomes across parasitic lice? (2) how often has mitogenome fragmentation evolved in lice, (3) is there evidence that mitogenome fragmentation is a non-adaptive process? and (4) does fragmentation result in lower AT% of mitogenomes? Altogether, this study considerably advances our understanding of the process of mitogenome fragmentation in parasitic lice, the implications of which can provide new insight into basic cellular and metabolic function across eukaryotes.

## Results

### Mitochondrial genome organization is highly variable in parasitic lice

**Fig. 1:**
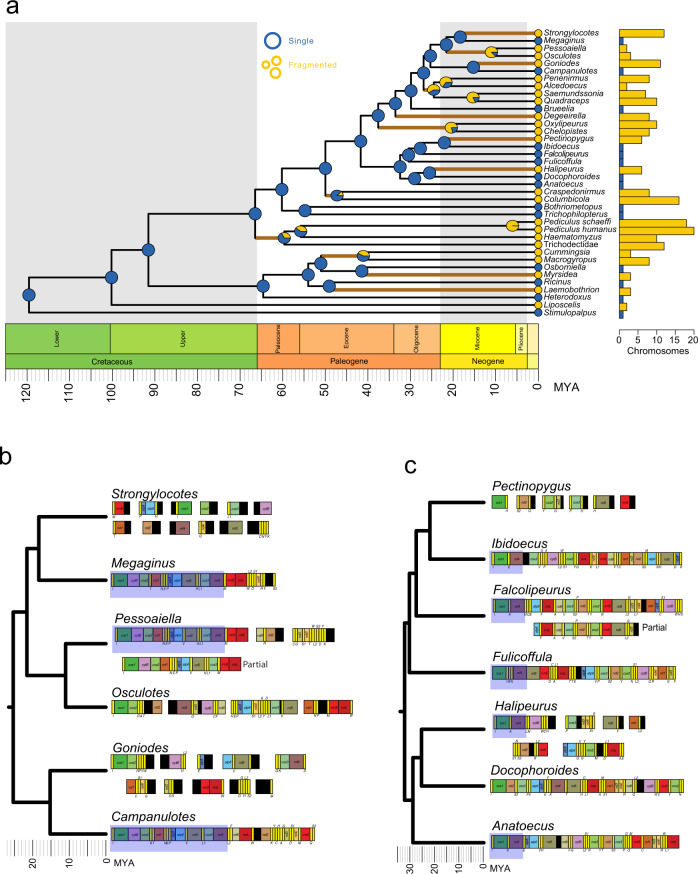
Phylogenetic patterns of mitochondrial genome structure in parasitic lice. **a** Dated phylogenetic tree of 36 taxa of parasitic lice and two outgroup taxa, representing all major clades of lice adapted from a nuclear gene phylogeny^28^ Circles at the tips indicate the mitogenome structure for that taxon: fragmented (yellow) or a single chromosome (blue). Pie charts at the internal nodes show the ancestral reconstruction of mitogenome structure, with the area of each color representing the relative likelihood of fragmented or single chromosomes. Brown branches indicate plausible transitions from single to fragmented mitogenomes and were used as foreground branches in tests for relaxed selection. Bar plot adjacent to the phylogeny shows the number of mitogenome fragments recovered for each taxon. **b**, **c** Details of the mitogenome structure for two clades of parasitic lice. Each chromosome has been arbitrarily (although consistently) linearized. Fragmented mitogenomes are shown with separate rectangles. Genes are colored as follows: *atp6* and *atp8*: shades of blue; *cox1-cox3*: shades of green; *cob*: purple; *nad1-nad6*: shades of brown; *rrnL* and *rrnS*: shades of red; tRNA genes: yellow. Conserved blocks of genes are highlighted by blue rectangles.

We assembled complete and partial mitogenomes from 24 genera of lice in Ischnocera (Table 1). Combined with previously published mitogenomes in lice (Supplementary Data 1), our comparisons revealed highly variable mitogenome organization in parasitic lice (Fig. 1a and b). Consistent with the previous studies^13,15,27^, these newly assembled mitogenomes have highly rearranged gene orders. Of 25 genera of Ischnocera (including new and previously published mitogenomes) 15 showed strong evidence for having more than one mitochondrial chromosome, 13 of which have (to the best of our knowledge) never been reported before. Most taxa with a fragmented mitogenome (all but *Halipeurus*) had at least one fragment with strong evidence for circularity based on read-mapping coverage and continuity (Supplementary Data 2). Most of the mitogenomes (all but three) are missing protein coding, rRNA, or tRNA genes (Supplementary Data 3), but these are all likely a result of incomplete assembly (either incomplete contigs or missing fragments). The level of mitogenome fragmentation varies considerably across the louse phylogeny (Fig. 1a). Among the 15 genera of Ischnocera with evidence for fragmented mitogenomes, the number of fragments ranges from three (*Pessoaiella*) to 16 (*Columbicola*)^20^. The lengths of different fragments in Ischnocera also vary depending on the genus, with complete fragments ranging from 1130 to 11430 bp (Supplementary Data 2).

**Table 1:** Summary of newly assembled mitogenomes from parasitic lice, including accession numbers for the original raw data used for assembly.

Louse species	Architecture	Number of chromosomes	GenBank Accession	NCBI SRA
*Campanulotes compar*	Single	1	ON643888	SRR5308113
*Alcedoecus* sp.	Fragmented	3	ON643882- ON643884	SRR5308110
*Anatoecus icterodes*	Single	1	ON643885	SRR5308111
*Brueelia antiqua*	Single	1	ON643886	SRR5308112
*Bothriometopus macrocnemis*	Single	1	ON643887	SRR5088466
*Chelopistes texanus*	Fragmented	8	ON643889- ON643896	SRR5308114
*Craspedonirmus immer*	Fragmented	7	ON643897- ON643903	SRR5308116
*Degeeriella rufa*	Fragmented	8	ON643904- ON643911	SRR5088467
*Docophoroides brevis*	Single	1	ON643912	SRR5308117
*Falcolipeurus marginalis*^a^	Single	1	ON643913	SRR5308118
*Fulicoffula longipila*	Single	1	ON643914	SRR5308119
*Goniodes ortygis*	Fragmented	10	ON643915- ON643924	SRR5308120
*Halipeurus diversus*	Fragmented	6	ON643925- ON643930	SRR5308124
*Ibidoecus bisignatus*	Single	1	ON643931	SRR5308126
*Megaginus tataupensis*^b^	Single	1	ON643932	SRR5308131
*Osculotes curta*	Fragmented	4	ON643933- ON643936	SRR5308133
*Oxylipeurus chiniri*	Fragmented	9	ON643937- ON643946	SRR5308134
*Pectinopygus varius*	Fragmented	6	ON643947- ON643952	SRR5308135
*Penenirmus auritus*	Fragmented	8	ON643953- ON643960	SRR5308137
*Quadraceps punctatus*	Fragmented	10	ON643961- ON643970	SRR5308139
*Saemundssonia lari*	Fragmented	7	ON643971- ON643977	SRR5308141
*Strongylocotes lipogonus*	Fragmented	12	ON643978- ON643989	SRR5308142
*Trichophilopterus babakotophilus*	Single	1	ON643990	SRR5308144
*Pessoaiella absita*^a^	Fragmented	3	ON643991- ON643993	SRR5308145

^a^Evidence for heteroplasmy.

^b^Missing several genes which are likely present on at least one other separate fragment.

**Fig. 2:**
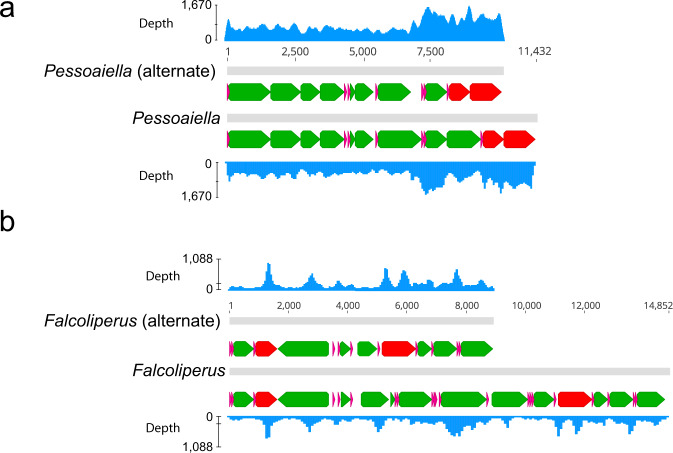
Evidence for heteroplasmic chromosomes in two species of lice. Heteroplasmic gene arrangements in (**a**) *Pessoaiella* and (**b**) *Falcoliperus*. The fragments are arbitrarily linearized to compare gene orders. The fragments are also shown with sequence coverage at each position. Genes are indicated with arrows (Green = protein coding gene; Green = rRNA; Pink = tRNA). Lengths (in base pairs) are indicated above each pair of fragments.

We also found evidence of heteroplasmy in mitogenome organization, defined here as multiple chromosome arrangements in an individual. *Pessoaiella* has three chromosome fragments, but one of the fragments has multiple arrangements. A longer fragment (~11,400 bp) contains the arrangement *cox3*-*nad4*-*trnM-rrnS* and a shorter fragment (~10,000 bp) is missing *nad4* with the arrangement *cox3*-*trnM-rrnS* (a 1269 bp deletion) (Fig. 2a). *Falcolipeurus* seems to possess a single full chromosome 14,839 bp, but we also found evidence for a smaller fragment that is missing a section containing the PCGs *nad1, atp8, cox1*, and *nad4* (a 5916 bp deletion) (Fig. 2b). Both heteroplasmic fragments had evidence for being circular molecules from AWA. Visual inspection of mapped reads in Geneious also showed consistent coverage (>500X in *Pessoaiella*; >80X in *Falcolipeurus*) across heteroplasmic regions and paired-end reads with pairs that spanned the 5′ and 3′ ends of the contigs.

### Fragmented mitochondrial genomes have evolved repeatedly in lice

To investigate the evolutionary history of mitogenome fragmentation in lice, we tested for phylogenetic signal of fragmented vs. single mitogenomes in lice using a published phylogenomic tree^28^. This phylogeny represents the most current and well-supported hypothesis of evolutionary relationships among parasitic lice. We combined information from mitogenomes assembled in the current study with previously sequenced mitogenomes to match this prior sampling, for a total of 36 louse taxa. Using an Equal-Rates (ER) model (AICc = 52.6), phylogenetic reconstruction favored fragmented mitogenomes as the ancestral state in lice, with at least eight transitions to single-chromosome mitogenomes (Supplementary Fig. 1). We also ran a trait reconstruction model that prohibited transitions from fragmented to single mitogenomes, which recovered single-chromosome mitogenomes as the ancestral state in lice and at least 13 transitions from single to fragmented mitogenomes (Fig. 1a). Although the AICc score for this directional model was slightly worse than that for the ER model (AICc = 53.7), we favor the directional model for several reasons. First, given that more distantly related outgroups not included in our reconstruction have a single mitochondrial chromosome^29,30^, it seems unrealistic that the common ancestor of bark lice, book lice, and parasitic lice (Troctomorpha) had a fragmented mitogenome, a scenario that is slightly favored in the ER model. Second, there are conserved blocks of gene arrangements within louse clades that were likely broken up by fragmentation. For example, *Megaginus, Pessoaiella*, and *Campanulotes* share a long block of 16 genes from *trnI* to *nad4* (although *Campanulotes* has three additional tRNA genes in this stretch). However, these three taxa do not form a clade, and the sister taxon of each has a highly fragmented or rearranged mitogenome (Fig. 1b). Third, the phylogenetic structure of specific clades of lice suggests the common ancestor of those clades possessed a single chromosome mitogenome. For example, *Brueelia* (single chromosome) is a sister to a clade consisting mostly of lice with fragmented mitogenomes. Finally, there are diverse clades of lice, for example, mammal lice, that all have highly fragmented genomes and there is no evidence of reversal to single chromosomes within these clades, suggesting that mechanistic reversal from a fragmented genome to a single chromosome could be extremely difficult.

### Mitogenome organization is stable within a species of louse, but heteroplasmic arrangements can be present

**Fig. 3:**
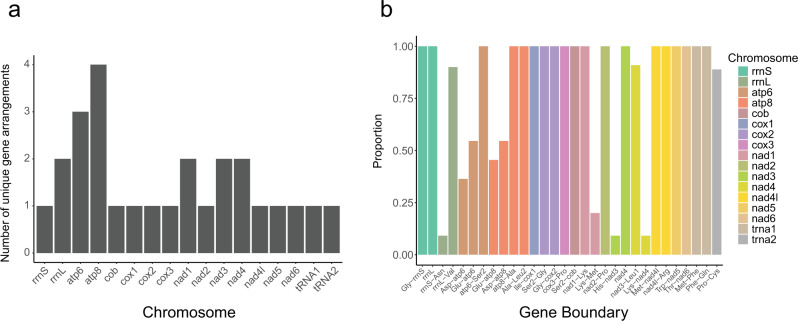
Unique gene boundaries of mitogenomes within a single species of louse. **a** Unique gene arrangements on the 17 chromosomes of mitogenomes from 11 individual lice within the species *Columbicola passerinae* 2. **b** Proportion of the 11 individual lice from *C. passerinae* 2 that possess a particular gene arrangement. Genes listed alone indicate only a single gene is present on that particular chromosome.

**Fig. 4:**
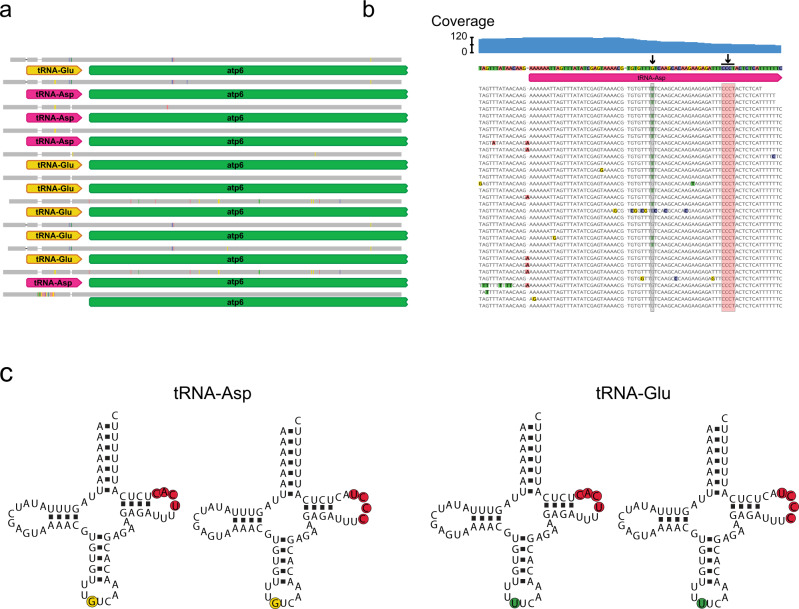
Variation in mitochondrial gene order and evidence of heteroplasmy within a single species of louse. **a** Alignment of the *atp6* gene and adjacent tRNA from 11 individual lice within the species *Columbicola passerinae* 2. Identical regions of the alignment are in gray and differences are shown as gaps (indels) or colored lines. **b** Section of an alignment of Illumina reads to the tRNA-Asp gene from a single individual of *C. passerinae* 2. Columns highlighted with boxes and arrows indicate regions that are variable among different individuals. The gray region shows a single nucleotide that has evidence of heteroplasmy (T or G), whereas the red region shows several nucleotides that alter tRNA secondary structure but do not appear to be heteroplasmic. **c** Four different tRNA structures among 11 individuals of *C. passerinae* 2. Nucleotides that vary among individuals are colored as follows: yellow =  G, green = T, red = four base indel.

To test whether mitogenome organization is variable within a species of louse, we assembled and annotated the mitogenomes from 11 individuals of *C. passerinae* 2 (the number reflects likely cryptic species diversity in *C. passerinae*^31^). These individuals were collected from four different host species and several geographic regions^23^. Mitogenome organization was mostly consistent across all 11 individuals of *C. passerinae* 2. We did initially find, however, that there were some differences in gene arrangements among the 11 individuals of *C. passerinae* 2 (Fig. 3a and b). Most of these differences involve the *tRNA-Glu* and *tRNA-Asp* genes on fragments containing *atp6* (three unique arrangements) and *atp8* (four unique arrangements) (Fig. 3a and b). The sequences of the two tRNA genes on the *atp6* and *atp8* fragments are mostly identical except for a single substitution that changes the anticodon from *Glu* to *Asp* or vice versa (Fig. 4a, c). However, mapping reads to the fragments containing these differences show that individual lice have high coverage for both the *Glu* and *Asp* anticodons at a single site (Fig. 4b). In other words, these gene rearrangements are likely not full rearrangements, but rather heteroplasmy within an individual louse. We also found no correlation between gene order and genetic distances within a single species of louse (*r* = 0.12, *p* = 0.42). In contrast, comparisons between species indicate consistent differences in gene order and sequence variation (Supplementary Figs. 2–3; Supplementary Data 4–5).

### Fragmented mitogenomes in lice are less AT-biased than the mitogenomes of other insects

**Fig. 5:**
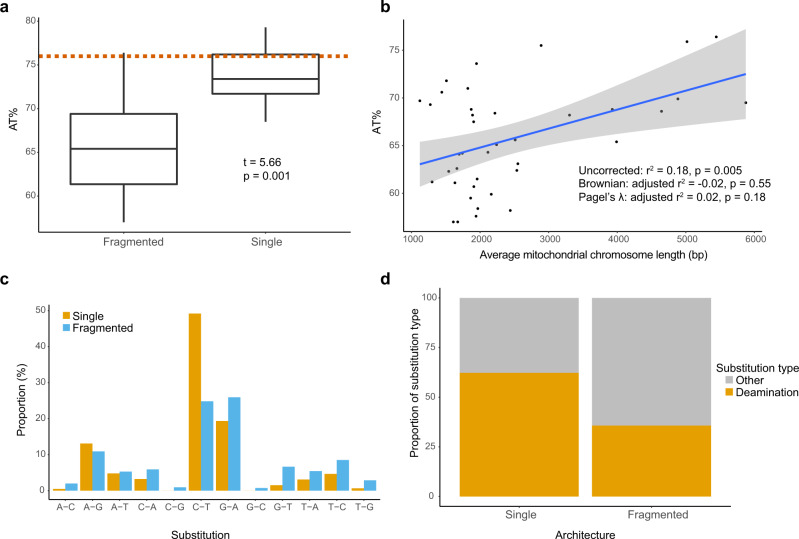
Patterns of AT% in fragmented versus single-chromosome mitogenomes in parasitic lice. **a** Box-and-whisker plot comparing AT% from fragmented and single-chromosome mitogenomes in lice (center line: median; box limit: upper and lower quartiles; whiskers: interquartile range). AT% calculated from coding and non-coding regions. Dotted red line shows the average AT% for insect mitogenomes available on NCBI GenBank (76.0%). Significance determined with phylogenetically corrected *t*-test. **b** Mitochondrial chromosome length is positively correlated with AT% in lice with fragmented mitogenomes. Points represent individual species of lice, where AT% is calculated from the total sequences and length is averaged among all chromosomes. The blue line and gray section indicate predictions from the linear model along with 95% confidence interval for the model. Results from statistical analyses, including from both phylogenetically corrected and uncorrected models, are indicated in the bottom right. **c** Proportion of substitutions among fourfold degenerate sites in the mitogenomes of 36 individuals (5 taxa) of *Columbicola* (fragmented mitogenome) and 35 individuals (7 taxa) of *Physconelloides* (single mitogenome). **d** Proportion of deamination substitutions (C → T and A → G) relative to other substitutions among fourfold degenerate sites in the mitogenomes of *Physconelloides* (single mitogenomes) and *Columbicola* (fragmented mitogenomes).

Consistent with previous studies^21,25^, we found that lice with fragmented mitogenomes have a significantly lower AT% (mean 65.8%) than lice with single mitogenomes (73.8%) (phylogenetically corrected post hoc test; *t* = 5.66, *p* = 0.001) (Fig. 5a). The nucleotide composition for lice with single mitogenomes (73.8%) is similar to that of most other insect mitogenomes (mean 76.0%). This pattern was consistent across coding regions, different codon positions, and fourfold degenerate sites (Supplementary Figs. 4–5; Supplementary Data 1). Lice in *Physconelloides* (single mitogenome) had a much higher rate of C → T mutations (49.1%) than *Columbicola* (fragmented mitogenomes) (24.8%), and a higher proportion of deamination mutations overall (*Physconelloides*: 62.3%; *Columbicola*: 35.7%) (Fig. 5c and d; Supplementary Data 6). Uncorrected linear models for both all sites and fourfold degenerate sites showed significant correlations between AT% and chromosome length in lice with fragmented mitogenomes (All sites: adjusted *r*^2^ = 0.18, *p* = 0.005; Four-fold sites: adjusted *r*^2^ = 0.08, *p* = 0.05) (Fig. 5b). However, phylogenetically corrected models did not show this correlation for either all sites (Brownian: adjusted *r*^2^ = −0.02, *p* = 0.55; Pagel’s λ: adjusted *r*^2^ = 0.02, *p* = 0.18) or fourfold degenerate sites (Brownian: adjusted *r*^2^ = −0.004, *p* = 0.29; Pagel’s λ: adjusted *r*^2^ = 0.013, *p* = 0.23) (Supplementary Fig. 6).

### Evolution of fragmented mitogenomes is associated with evidence for relaxed selection in mitochondrial genes

There was a weak but significant signal of relaxed selection (i.e., *dN/dS* ratios towards 1.0, indicating less stringent purifying selection) along the test (transitions from single to fragmented mitogenomes) branches relative to the background branches using both nuclear (*k* = 0.94, *p* = 0.007, LR = 7.26) and mitochondrial (*k* = 0.95, *p* = 0.012, LR = 6.27) phylogenies. In both analyses, most codon sites increased towards 1 along test branches, indicating most sites are under stronger purifying selection in background branches (Supplementary Figs. 7–8).

## Discussion

Comparisons of the mitochondrial organization across the diversity of parasitic lice (Phthiraptera) revealed that fragmented mitogenomes have evolved from single chromosome mitogenomes many times in the group (Fig. 1a). This pattern of independent origins of fragmented mitogenomes is consistent with previous work^13,20,21^, but our study demonstrates that fragmentation is much more widespread and has evolved more frequently than was previously assumed for this group of insects. Our data set includes mitogenomes from all major lineages of parasitic lice, so our findings provide a broad picture of the evolution of mitogenome structure within the group, particularly at deeper evolutionary scales. These structural transitions towards fragmentation are associated with signatures of relaxed selection in mitochondrial protein-coding genes (*dN/dS* ratios shifting towards 1.0) (Supplementary Figs. 7–8) and with decreases in AT% (Fig. 5a; Supplementary Data 1), which provide some key insights into the causes and consequences of mitogenome fragmentation.

High AT% in the mitogenomes of insects (and other animals) is thought to be mainly caused through G → A mutations or the occurrence of deamination mutations, especially C → T mutations^26^. During replication, the sense strand can be left unbound for up to two hours, leaving the strand particularly exposed to deamination mutations^32^. In fragmented mitogenomes, replication would theoretically take less time, thus limiting the exposure time for deamination mutations to occur relative to larger, single chromosome mitogenomes. Consistent with this idea, some of our results showed considerably more C → T mutations in a group of lice with single chromosome mitogenomes (*Physconelloides*) compared to a similar group with fragmented mitogenomes (*Columbicola*) (Fig. 5c and d; Supplementary Data 6). Because fragmented mitogenomes of lice have less of a mutation bias but also have a higher overall mutation rate compared to single-chromosome mitogenomes, fragmented mitogenomes could shift to a more balanced nucleotide composition in a relatively short amount of time.

Relaxed selection (or reduction in the efficiency of selection) in conjunction with high rates of mutation suggests that fragmentation could be a nonadaptive process (although see discussion below on a selective model). This would be consistent with other systems, including the highly fragmented genomes of endosymbiotic bacteria of cicadas^33^ and large mitogenomes of certain plants^34^. However, relaxed selection in mitochondrial PCGs alone does not explain why some lice have fragmented mitochondrial genomes and others do not. It may also be the case that the nuclear genome in lice overall is subject to relaxed selection, as suggested by their simplified morphology (e.g., reduced eyes, lack of wings, etc.), and this could lead to relaxed selection for mutation repair. Given that many mitochondrially targeted genes, including those involved in mitochondrial replication, are encoded by the nuclear genome, some of these genes may have experienced knockout mutations (such as mitochondrial single-stranded binding protein—*mtSSB*)^13^ resulting in increased mitochondrial mutation rates. Under this scenario, whether a lineage or clade of lice has fragmented mitogenomes is determined by the random loss of particular nuclear genes.

Despite considerable variation in mitogenome organization among different louse taxa, we found stable organization within a single species (Fig. 3a and b; Supplementary Figs. 2–3; Supplementary Data 4–5), and any differences in gene order seem to be from heteroplasmic variation at an anti-codon site, resulting in alternative tRNA genes (Fig. 4a–c). We also found evidence for more extreme cases of heteroplasmy, where an individual louse can have multiple types of chromosomes (e.g., a larger chromosome and another, smaller chromosome containing a subset of genes) (Fig. 2). Mitochondrial heteroplasmy has become documented with increasing frequency in many different organisms (including lice^22^), and can result from a lack of sorting of mitochondrial mutations within the germ line or paternal leakage of mitochondrial DNA^35,36^. The majority of these cases are differences at single sites or in gene lengths, and although there are instances of deleterious heteroplasmic alleles^13,22^, it is seemingly rare for heteroplasmy to result in changes in gene order.

Heteroplasmy in mitogenome organization may also provide a clue for a potential mechanism for fragmentation to initiate and become more extreme. Within the nematode *Caenorhabditis briggsae* some strains have a heteroplasmic copy of a partial mitochondrial genome that has a large deletion (including the *nad5* gene) compared to the full chromosome^37^, similar to the situation in some lice. The frequency of the heteroplasmic strain can increase in experimental populations, even though possessing the heteroplasmic chromosome is deleterious. This increase occurs through the biased transmission of the heteroplasmic chromosome in which it acts as a selfish genetic element^37^. A similar process could be occurring in lice. If a heteroplasmic fragment containing a large deletion arises in a lineage (perhaps because of reduced efficiency of mitochondrial replication machinery), this fragment could initially persist through biased transmission. If the original full chromosome then experiences a knock out mutation of an essential gene, but one which was still intact on the heteroplasmic fragment, this would then maintain the fragment through strong selection, because without the fragment the knock out mutation would be lethal. In this scenario, the original chromosome is also retained, because it contains other essential genes that do not occur on the heteroplasmic fragment. Further knock out and deletion mutations on either chromosome might even be selected for, as long as they do not knock out a gene contained on only one of the two chromosomes. This process could continue until both chromosomes in aggregate contain only a single copy of each gene, resulting in two chromosomes both maintained by strong selection. This cycle could repeat if new heteroplasmic fragments of the two smaller chromosomes arise. This process could also continue until each chromosome contains only a single or very few genes, similar to the situation seen in mammal lice and some bird lice. Such a process could also explain why relaxed selection (and in particular an upward shift in dN/dS) is associated with transitions to fragmented mitogenomes in lice, but under a selective model (rather than a nonadaptive drift model, see discussion above). When both chromosomes are in the stage where they each contain a copy of the same functional gene, both copies might accrue amino acid replacements more readily than if only a single copy of the gene existed. Once a knock out mutation occurred, the lineage would be “stuck with” the only functional copy, but one that has now accrued many amino acid replacement mutations. Further evidence for this possible mechanistic pathway could be obtained by examining in more detail clades of lice that differ in mitogenome fragmentation by uncovering whether there is a stepwise process consistent with a heteroplasmic fragment as an intermediate step.

Our results also highlight the need for future work on different taxonomic and evolutionary scales. For example, it could be that fragmentation has an underlying genetic component conserved as far back as the common ancestor of Acercaria (lice, thrips, true bugs, and relatives). On a smaller evolutionary scale, our results suggest that investigations within specific clades of lice will likely be fruitful for further understanding mitogenome fragmentation. For example, comparisons among many louse taxa with variable mitogenome structures in a clade could allow for a more detailed reconstruction of the process and direction of fragmentation. Working with more closely related taxa could also help clarify the nature of relaxed selection. Although we found a significant signal of relaxed selection associated with fragmentation, the signal was weak overall, likely because the taxa in our data set are highly divergent. Despite these limitations, our findings are a crucial development in our understanding of mitogenome fragmentation in lice. Our results help establish the broader patterns of mitogenome structure across parasitic lice, provide evidence for a selective explanation for fragmentation, demonstrate a shift in nucleotide composition as a likely consequence of fragmentation, and build a foundation for future research that explores this molecular phenomenon in lice and other insects.

## Methods

### Mitogenome assembly and annotation

We assembled the mitogenomes from existing genomic data of 24 genera in the louse Parvorder Ischnocera^28^. These data are whole-genome shotgun, 160 bp paired-end reads generated from an Illumina HiSeq2500 with insert sizes of ~400 bp. The samples were extracted from single or pooled (between 3–50) individuals collected from wild or captive birds. Before assembly, we trimmed adapters and low-quality bases using Trimmomatic v.0.36^38^ (leading and trailing bases below a quality of 3, 4-base sliding window below a quality of 15) and removed reads <75 bp. We then checked the qualities of the trimmed reads using FastQC v.0.11.7 (Babraham Bioinformatics). To assemble the mitogenomes, we followed previously developed pipelines that use a combination of targeted, de novo, and read mapping assembly^20^. First, we assembled protein-coding genes (PCGs) for each species using aTRAM v.2.3.1^39^. We converted the trimmed paired-end libraries into BLAST-formatted databases with 50 shards, and assembled the genes using ABySS v2.2^40^ as the de novo assembler, 3 iterations, 10% of the library, and protein sequences of mitochondrial PCGs from the published *Campanulotes compar* mitogenome as targets^13^. Because mitochondrial reads are much more prevalent than nuclear reads in shotgun sequence data, using 10% of the library ensures against assembling sequencing errors or nuclear mitochondrial DNA (numts), while also having more than adequate coverage for confident assemblies (often >100X)^20^. We also attempted to assemble mitochondrial sequences using a completely de novo approach using SPAdes v.3.11.1^41^, using the default parameters and 10% of the reads. We identified mitochondrial assemblies by BLASTing scaffolds against published mitochondrial gene sequences from either *C. compar* or *Ibidoecus bisignatus*. However, de novo assemblers can have difficulties assembling through the non-coding control regions of mitogenomes, primarily because of repeats or partially conserved sequences between different genome fragments^42^. To avoid this issue and assemble full mitogenomes, we used the iterative-mapping approach in MITObim v.1.8^43^. For each trimmed library, we ran MITObim with the –quick option using each assembled gene (from aTRAM) or contig (from SPAdes) as a starting reference. As with the de novo assemblies, we used 10% of the trimmed read libraries for each MITObim run. We then created majority consensus sequences from the MAF files using the “miraconvert” command in MIRA v.4.0.2^44^. We combined these outputs using the “Medium-Low Sensitivity” setting in the Geneious de novo assembler in Geneious Prime v. 2020.1.2 (Biomatters, Ltd.). We considered the resulting contigs as candidate complete mitochondrial chromosomes. We tested for the circularity of these assemblies using AWA^45^, which splits a contig, flips the two halves, and maps paired-end reads to the adjacent 5’ and 3’ ends of the contigs to test for circularity. We considered strong evidence for complete circularity to have a match score >95%, an alignment score > −2, and continuous connection coverage >20× across the 5′ and 3′ ends of an assembled contig. Finally, we annotated each assembly using the MITOS2 webserver^46^, with subsequent manual adjustments using open reading frame information and compared against published mitochondrial genes from *Campanulotes compar* or *Ibidoecus bisignatus*^13^.

### Ancestral reconstruction

To reconstruct the ancestral patterns of mitogenome structure (fragmented vs. single-chromosome) in parasitic lice, we used an existing phylogeny based on nuclear data and mitogenome information from 36 ingroup and 2 outgroup taxa (the 24 mitogenomes from this study combined with existing mitogenomes)^28^. We tested three different models of ancestral reconstruction: an Equal-Rates (ER) model, an All-Rates-Different (ARD) model, and a custom model that did not allow for transitions from fragmented mitogenomes to a single mitogenome. We assessed model fit with the corrected Akaike Information Criterion (AICc). We ran ancestral state reconstructions with the *ace* function in the APE v.5.4^47^ package in R and assessed model fit with the *fitDiscrete* function in the GEIGER v.2.0.7^48^ R package.

### Intraspecies comparison

To compare mitogenome structure and arrangements in a single species of louse, we used data from the dove louse taxon *Columbicola passerinae* 2 (the number reflects likely cryptic species diversity in *C. passerinae*)^23,31^. We assembled the mitogenome fragments from 11 individuals of *C. passerinae* 2 with aTRAM v.2.3.1^39^, using genes from the mitogenome of *C. passerinae* 2^20^ as targets, and running aTRAM with 10 iterations, between 10% and 100% of the libraries, and ABySS v.2.2^40^ for de novo assembly. We then annotated the assembled contigs using MITOS2. We included data from two other species as outgroups (*C. passerinae* 1 and *C. columbae*). We then compared the differences in gene order among the lice using a presence/absence matrix of gene boundaries. To compare the distance matrix of gene boundaries to genetic distances, we calculated uncorrected and corrected (JC and K80) genetic distances from the mitochondrial gene sequences using the *dist.dna* function in APE. We then compared the gene boundary and genetic distance matrices with Mantel Tests (999 permutations) using the *mantel.randtest* function in the ade4 v. 1.7^49^ R package.

For any variable gene arrangements within *C. passerinae* 2, we used Bowtie2 v.2.3.5.1^50^ to map 10% of the paired-end reads against the mitogenome fragments to test for A) validation of assemblies and B) evidence for heteroplasmy (i.e., multiple gene arrangements within an individual louse). We viewed resulting read alignment BAM files in Geneious Prime v.2020.1.2 (Biomatters, Ltd.) to assess coverage and screen for sequence variants associated with variable gene arrangements. Finally, we compared tRNA secondary structure with the Vienna package RNAfold (http://www.tbi.univie.ac.at/~ivo/RNA/RNAfold.html) in Geneious for fold prediction, using the Turner energy model^51^ rescaled to 37 °C. We confirmed these inferred structures by eye in comparison to those inferred for *Bothriometopus*^52^.

### Nucleotide composition

We tested for differences in nucleotide composition in all available full mitogenomes of parasitic lice (as of October 2020; Supplementary Data 1). This dataset included the sequences from the current study in addition to sequences available on NCBI GenBank (8 Ischnocera, 10 Amblycera, 4 Trichodectidae, 1 Rhynchophthirina, and 12 Anoplura). From these data, we calculated AT% in seven subsets of the data: all sites, coding regions, different codon positions, and fourfold degenerate sites in PCGs (Supplementary Data 1). We tested for significant differences in AT% among the different subsets using corrected *t*-tests in the GGPUBR v. 0.40R package^53^.

Before comparing AT% between different groups of lice, we first estimated phylogenetic relationships among the lice to account for phylogeny in our comparisons. We extracted mitochondrial PCGs from the full mitogenomes, translated each gene to amino acids, and aligned them using the –auto option in MAFFT v.7^54^. We included sequences from *Liposcelis sculptilimacula*^55^ as an outgroup. We concatenated the gene alignments and estimated a maximum likelihood (ML) phylogeny using IQTree v.2.1.1^56^ (Supplementary Fig. 9). We tested for optimal partitions and amino acid substitution models using ModelFinder^57^. We then tested for significant differences in AT% between lice with fragmented versus single-chromosome mitogenomes using *t*-tests and accounting for phylogeny with phylogenetic ANOVA^58^ using the *phylANOVA* function in the PHYTOOLS v.0.7R package^59^. To test for a positive correlation between AT% and mitogenome fragment length, we fit linear and Phylogenetic Least Squares (PGLS) models to the louse mitogenome dataset. For lice with fragmented mitogenomes, we used the average length among the different fragments. We fit models using both total AT% and AT% of fourfold degenerate sites. For PGLS, we used the ML tree estimated previously in the *pgls* function from the CAPER v.1.0.1R package, with both Brownian and Pagel’s λ correlations.

We also wanted to test the hypothesis that there will be more C to T substitutions in fragmented mitogenomes compared to single mitogenomes. To do this, we used the mitochondrial data from refs. ^23,60^. These data are subsets of mitochondrial PCGs from lice in the genus *Columbicola* (5 taxa, 36 samples) and *Physconelloides* (7 taxa, 35 samples). *Columbicola* have highly fragmented mitogenomes^20^, whereas *Physconelloides* have a single mitogenome. We aligned the genes according to amino acids and extracted fourfold degenerate sites. We then estimated substitution rate matrices for these sites in MegaX v.10.1.8^61^ using GTR models and Γ distributions with four categories.

### Test for relaxed selection

We tested for evidence of relaxed selection associated with transitions from single to fragmented mitogenomes using RELAX in the HyPhy package^62^. RELAX tests whether dN/dS ratios significantly shift toward 1 (a signal of neutrality) in specific phylogenetic branches relative to the other background branches. We first identified branches involved in these transitions based on our ancestral state reconstructions (described above), using the transition branches as test branches and the remaining branches as references (Fig. 1). We ran RELAX using the 13 mitochondrial PCGs, and both the mitochondrial phylogeny estimated in the current study and the published nuclear phylogeny^28^, with the mitochondrial phylogeny trimmed to match the taxonomic representation of the nuclear phylogeny (36 taxa). We ran all RELAX tests on the Datamonkey webserver^63^.

### Reporting summary

Further information on research design is available in the Nature Research Reporting Summary linked to this article.

## Supplementary information

Supplementary Information

Description of Additional Supplementary Files

Supplementary Data 1

Supplementary Data 2

Supplementary Data 3

Supplementary Data 4

Supplementary Data 5

Supplementary Data 6

Reporting Summary

## Data Availability

Data associated with this study are available in the Supplementary material, NCBI GenBank (ON643882-ON643993), NCBI SRA, or on the Dryad Digital Data Repository (doi.org/10.5061/dryad.9w0vt4bhx)^64^. Other data are available on reasonable request.

## Code availability

Code used for generating results for this study are available on GitHub (https://github.com/adsweet/mitogenomes).
