# Supplementary material for: Independent evolution of highly variable, fragmented mitogenomes of parasitic lice
Source: Commun Biol. 2022 Jul 8;5:677. doi: 10.1038/s42003-022-03625-0 (PMC9270496; doi:10.1038/s42003-022-03625-0)
Supplement: Supplementary file 1 — Supplementary Information [file 42003_2022_3625_MOESM1_ESM.pdf]

Supplementary Information for

Independent evolution of highly variable, fragmented mitogenomes of parasitic lice

Andrew D. Sweet[1*], Kevin P. Johnson[2], and Stephen L. Cameron[3]

[1] Department of Biological Sciences, Arkansas State University, State University, AR, 72467 USA

[2] Illinois Natural History Survey, Prairie Research Institute, University of Illinois, Champaign, IL 61820 USA

[3] Department of Entomology, Purdue University, West Lafayette, IN 47907 USA

* Corresponding author: Andrew D. Sweet, asweet@astate.edu, P.O. Box 599, State University, AR 72467

SUPPLEMENTARY FIGURES

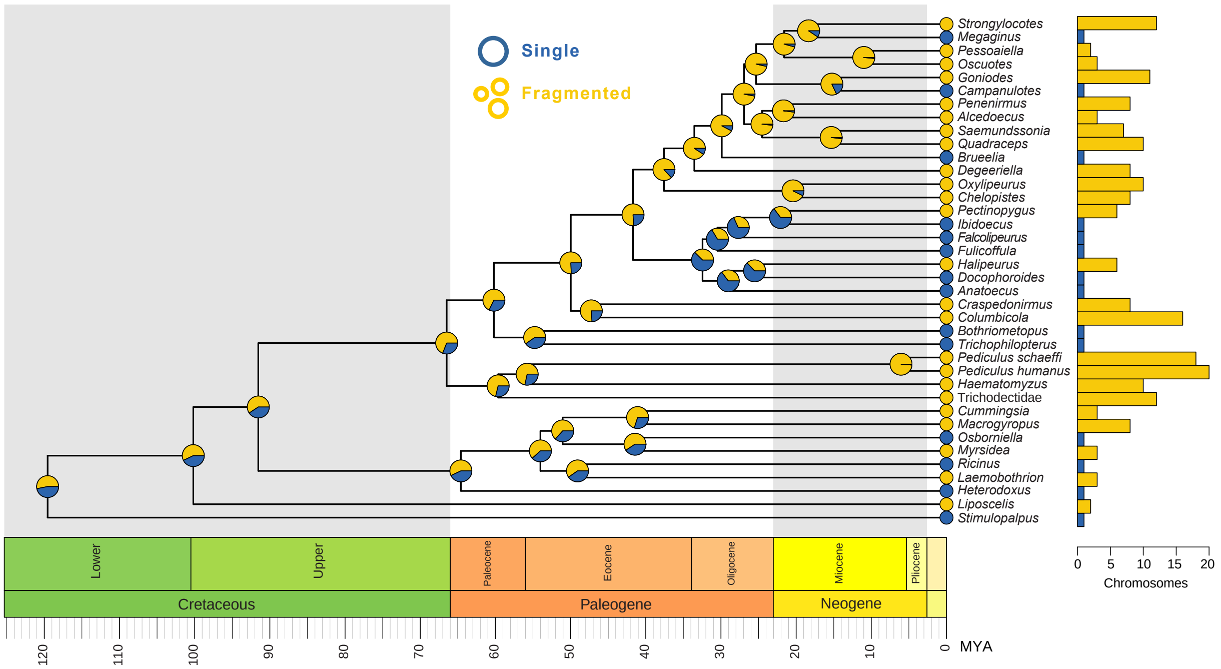

Supplementary Figure 1. Dated phylogenetic tree of 36 taxa of parasitic lice and two outgroup taxa, representing all major clades of lice. Phylogeny adapted from Johnson et al. (2018). Circles at the tips indicate the mitogenome structure for that taxon: fragmented (yellow) or a single chromosome (blue). Pie charts at the internal nodes show the ancestral reconstruction of mitogenome structure using an Equal Rates (ER) model, with the area of each color representing the relative likelihood of fragmented or single chromosomes. Brown branches indicate plausible transitions from single to fragmented mitogenomes and were used as foreground branches in tests for relaxed selection. Bar plot to the right of the phylogeny shows the number of mitogenome fragments recovered for each taxon. Some taxa have incomplete mitogenomes; in these cases the number of chromosomes are a minimum number.

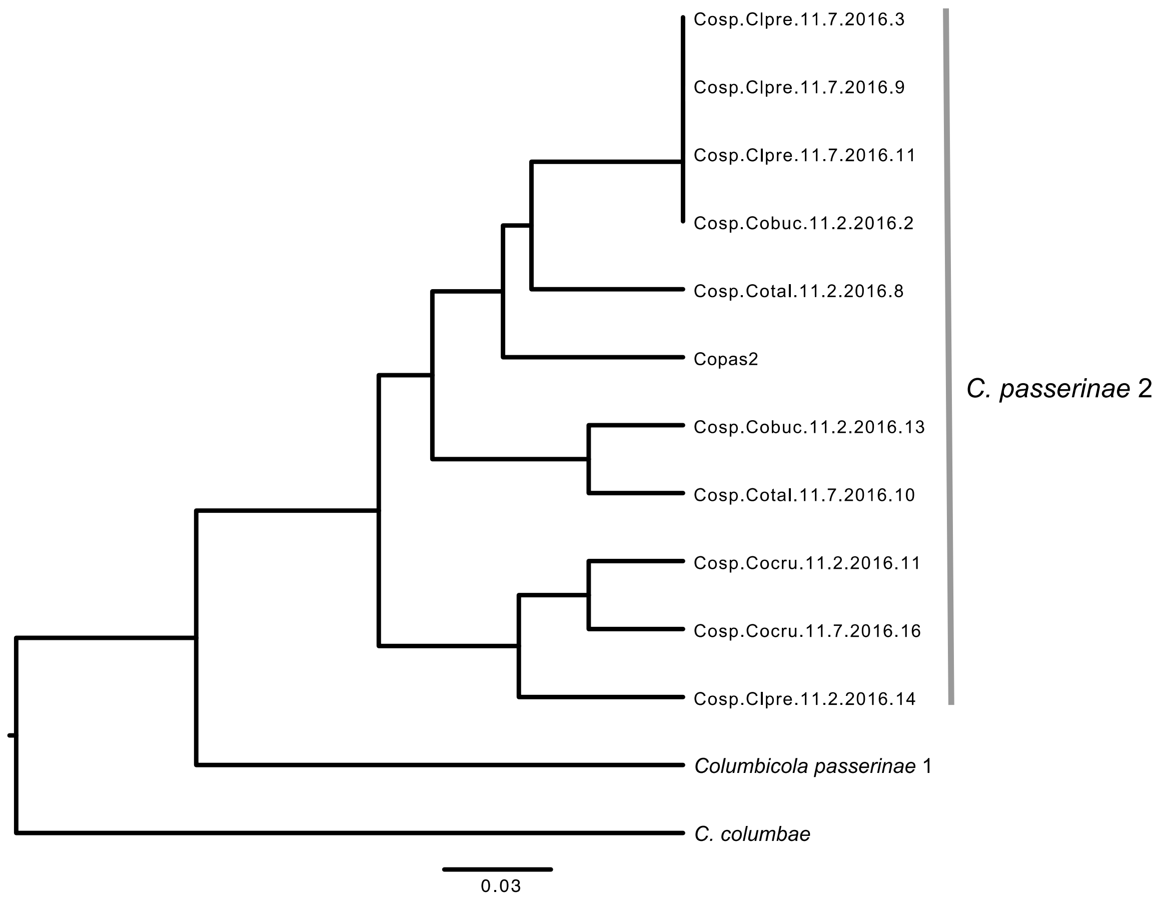

Supplementary Figure 2. UPGMA distance tree based on the order of genes (presence/absence of gene adjacencies) in the mitogenomes of 11 individuals from the species *Columbicola passerinae* 2 and two outgroup species: *C. passerinae 1* and *C. columbae*. Scale bar indicates distances.

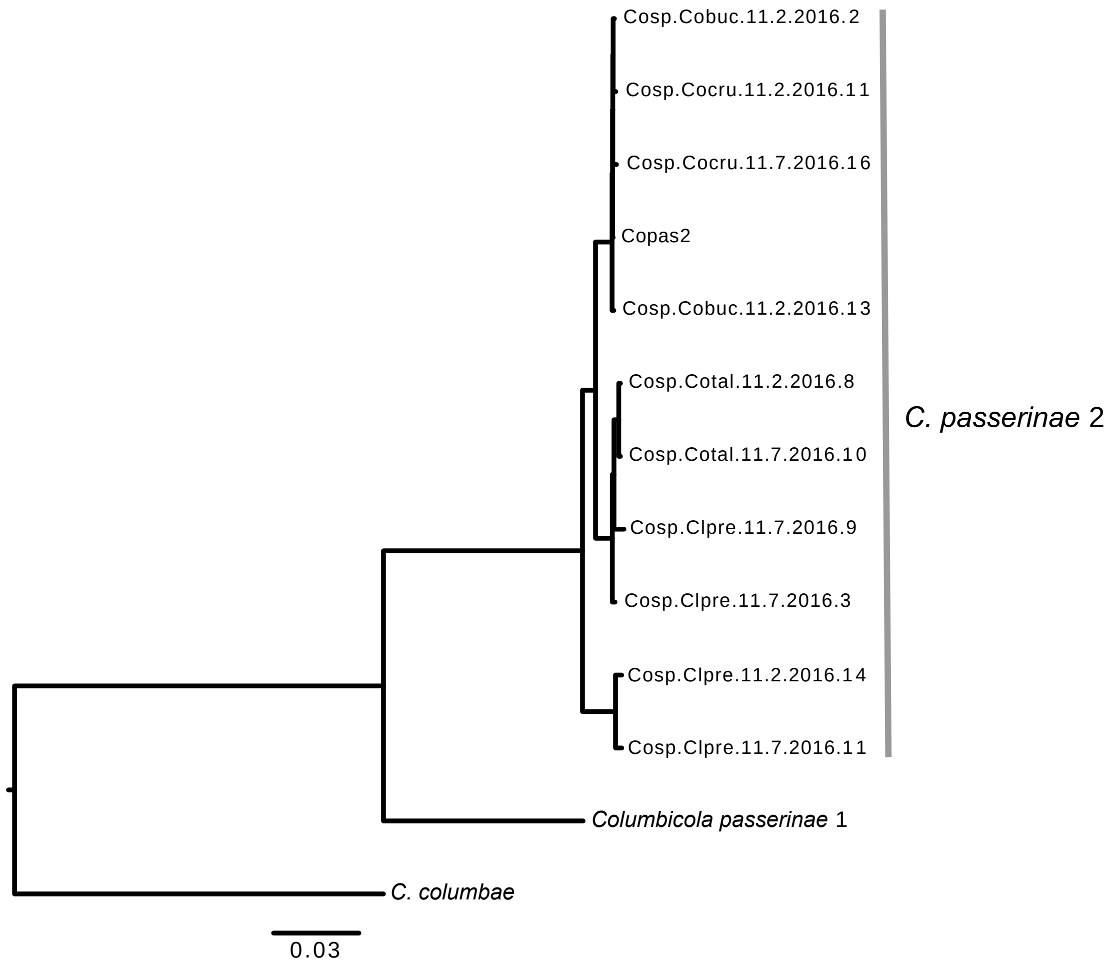

Supplementary Figure 3. Neighbor-joining tree based on the nucleotide sequences of genes in the mitogenomes of 11 individuals from the species *Columbicola passerinae* 2 and two outgroup species: *C. passerinae 1* and *C. columbae*. Scale bar indicates distances.

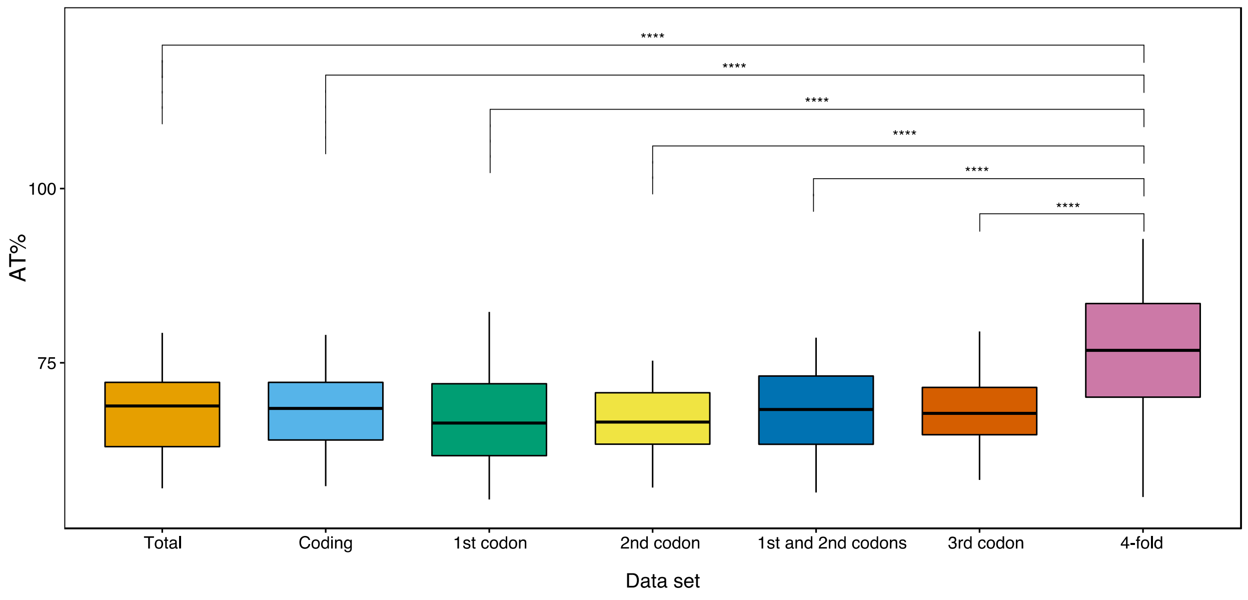

Supplementary Figure 4. AT% from different partitions of the mitogenomes from parasitic lice, including all sequences, coding regions, different codon sets, and 4-fold degenerate sites. Partitions with significantly different AT% are shown with brackets (**** = $p < 0.001$).

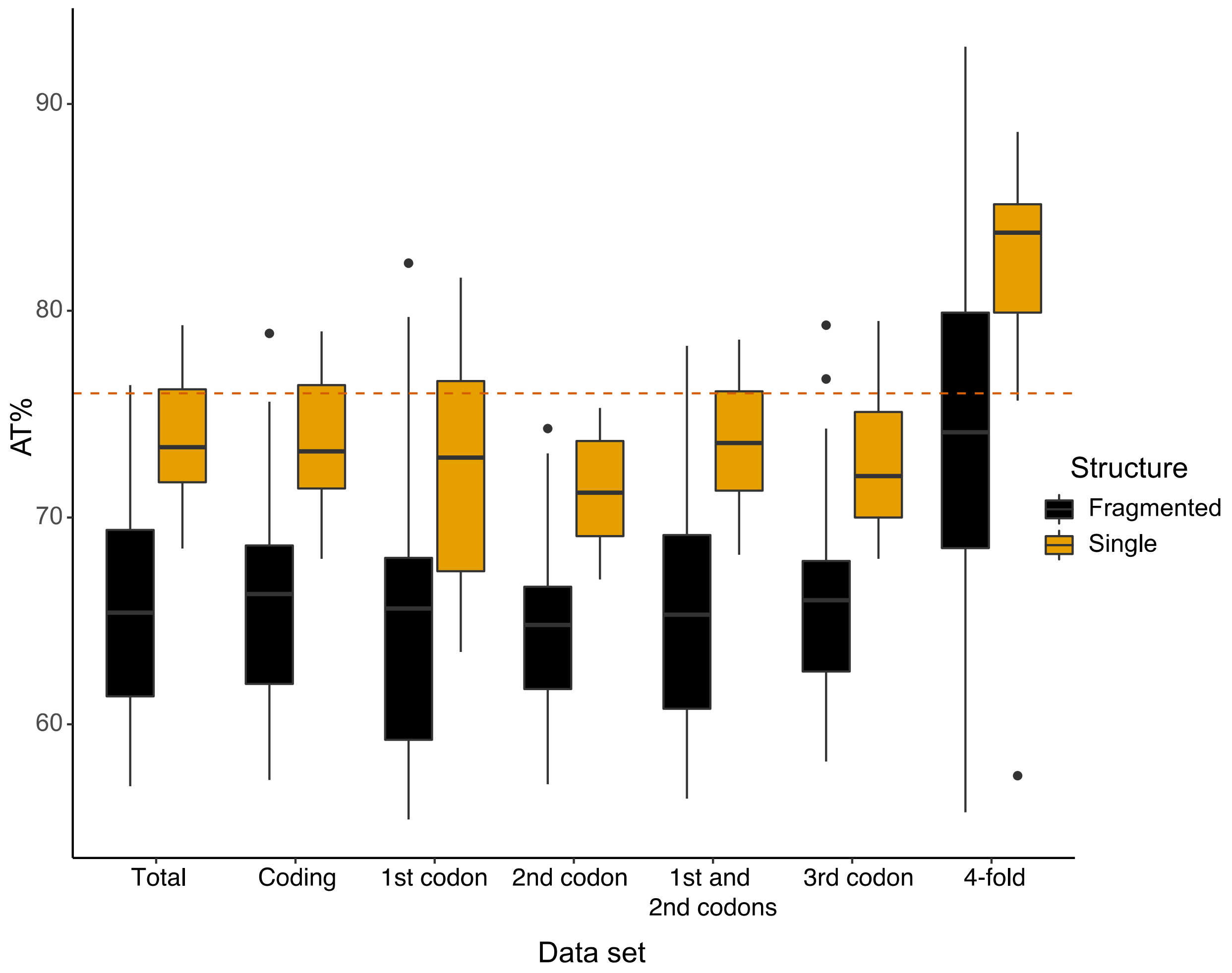

Supplementary Figure 5. AT% from different partitions of the mitogenomes from parasitic lice, including all sequences, coding regions, different codon sets, and 4-fold degenerate sites. For each partition, AT% is shown for lice with fragmented mitogenomes compared to those with a single chromosome. The red dotted line indicates the average AT% for the mitogenomes (all sequences) of other insects available in NCBI GenBank.

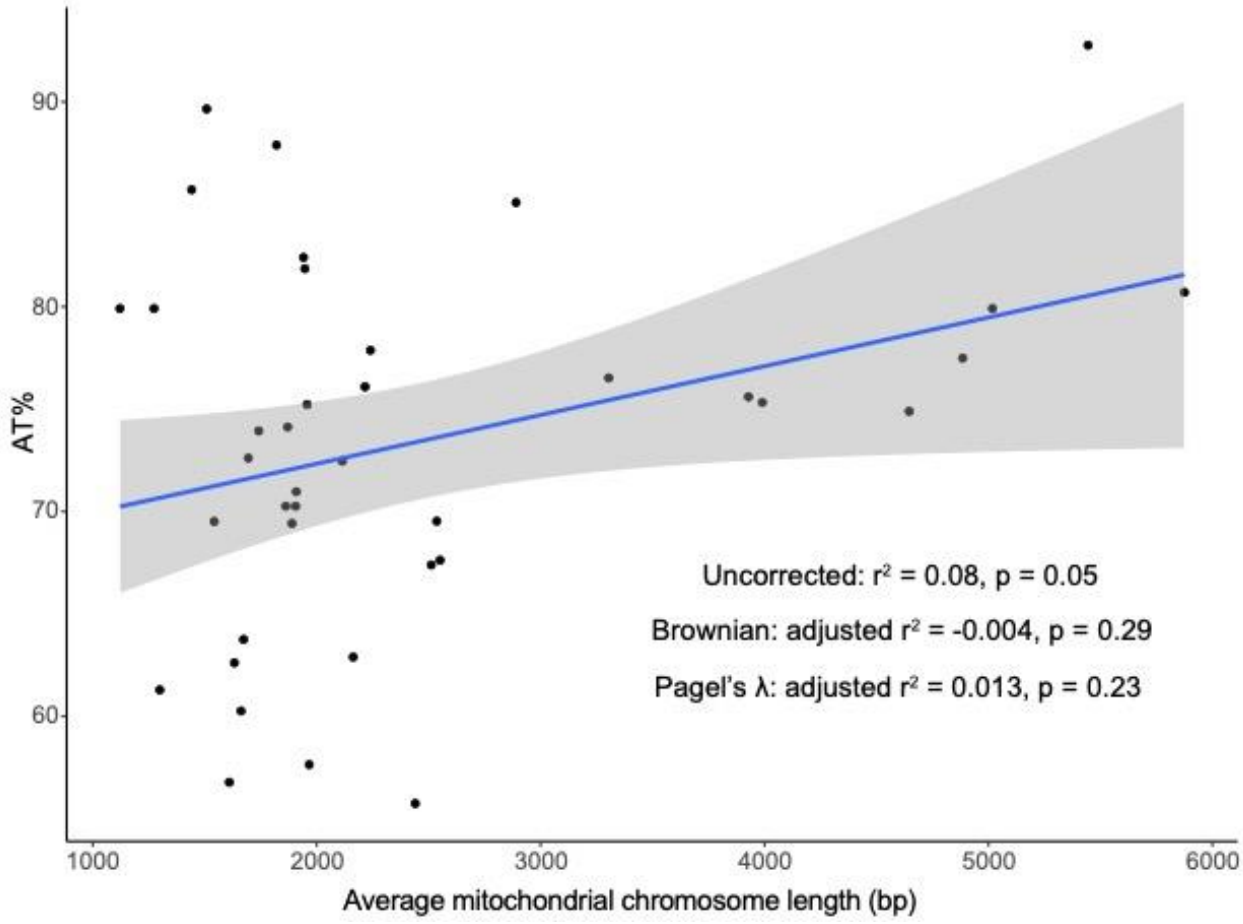

Supplementary Figure 6. Correlation between AT% and chromosome length in the mitogenomes of parasitic lice with fragmented mitogenomes. Points represent individual species of lice, where AT% is calculated from 4-fold degenerate sites and length is averaged among all chromosomes. The blue line and gray section indicate predictions from the linear model along with 95% confidence interval for the model. Results from statistical analyses, including from both phylogenetically corrected and uncorrected models, are indicated in the bottom right.

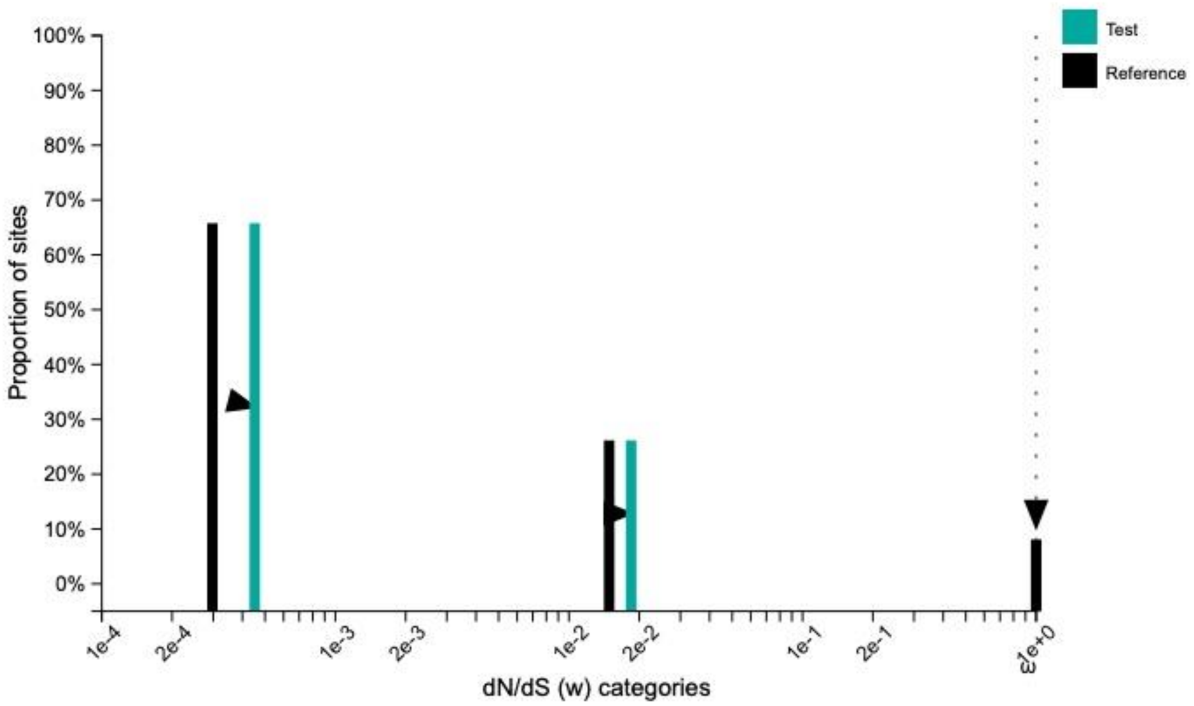

Supplementary Figure 7. dN/dS ratios from the mitochondrial protein coding genes (PCGs) of 36 species of parasitic lice. The sites are assigned to three categories of dN/dS ratios representing highly deleterious, moderately deleterious, and moderate positively selected or neutral sites. The different bars show proportion of sites from branches associate with transitions from single to fragmented mitogenomes ("Test") versus all other branches ("Reference") in a phylogenetic tree based on mitochondrial PCGs.

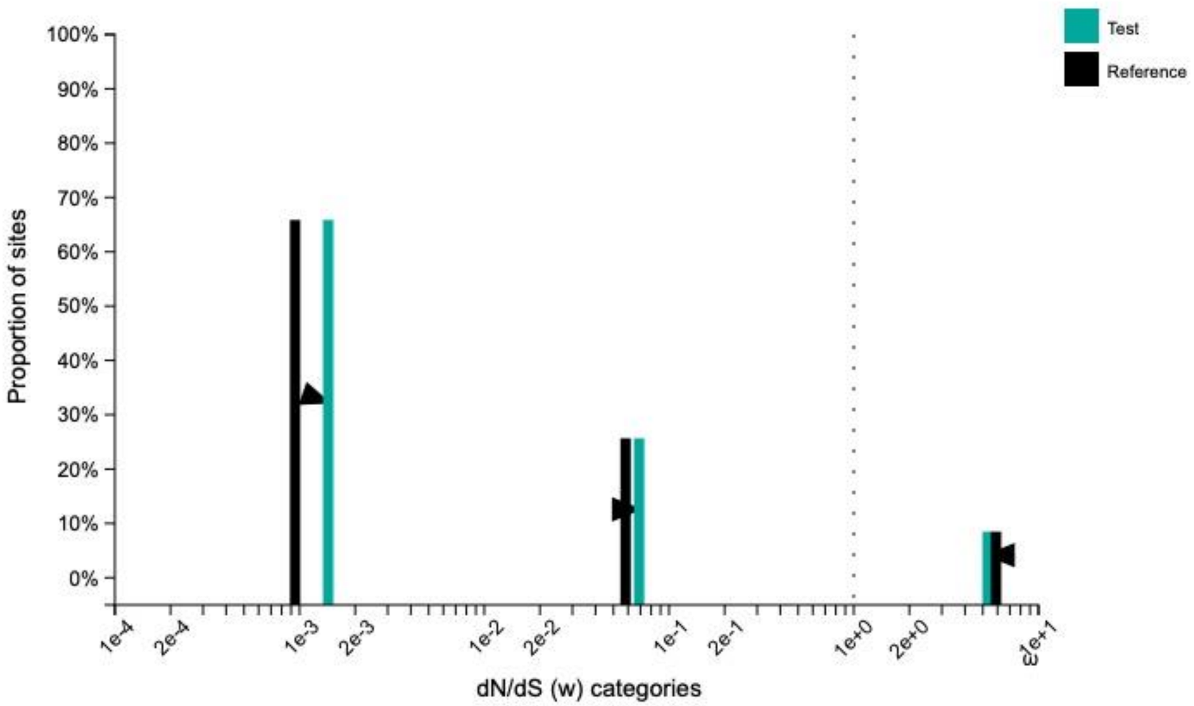

Supplementary Figure 8. dN/dS ratios from the mitochondrial protein coding genes (PCGs) of 36 species of parasitic lice. The sites are assigned to three categories of dN/dS ratios representing highly deleterious, moderately deleterious, and moderate positively selected or neutral sites. The different bars show proportion of sites from branches associate with transitions from single to fragmented mitogenomes ("Test") versus all other branches ("Reference") in a phylogenetic tree based on >1,000 nuclear genes.

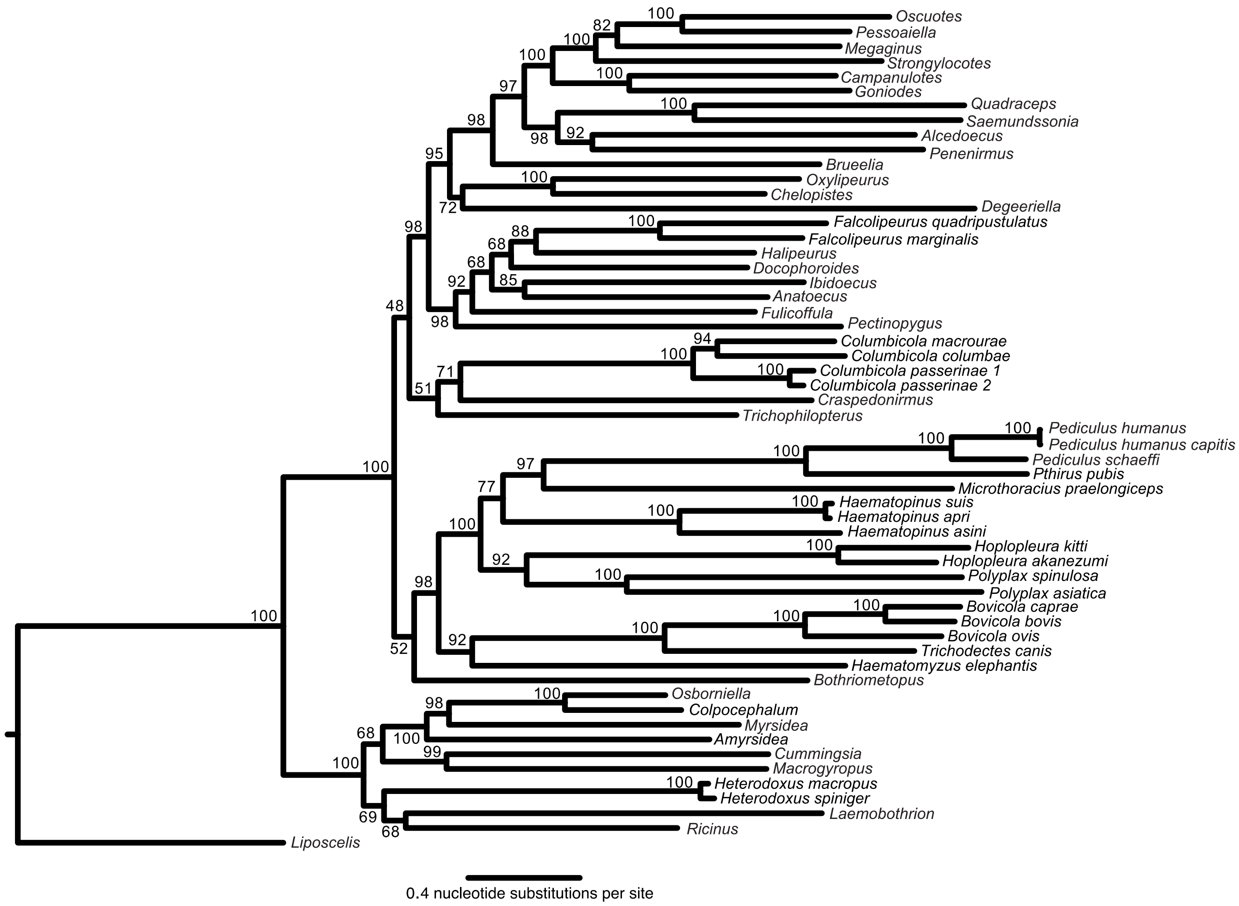

Supplementary Figure 9. Maximum likelihood phylogeny for parasitic lice based on amino acid sequences of mitochondrial protein coding genes. Sequence data obtained from existing or novel mitochondrial genomes of lice (Table S1). Bootstrap support values are indicated above each branch. Phylogeny is rooted on the book louse *Liposcelis*.
