# Supplementary material for: Independent evolution of highly variable, fragmented mitogenomes of parasitic lice
Source: Commun Biol. 2022 Jul 8;5:677. doi: 10.1038/s42003-022-03625-0 (PMC9270496; doi:10.1038/s42003-022-03625-0)
Supplement: Supplementary file 2 — Description of Additional Supplementary Files [file 42003_2022_3625_MOESM2_ESM.pdf]

Description of Additional Supplementary Files

File name: Supplementary Data 1
Description: Nucleotide composition, structure, and NCBI GenBank information for the mitogenomes of parasitic lice.

File name: Supplementary Data 2
Description: Assembly statistics for mitogenomes of 24 species of parasitic lice. Each chromosome is labeled according to the protein coding and ribosomal genes contained on that chromosome. Chromosomes that only contain tRNAs are also indicated. Match, Average Coverage, Average Connection, Continuous connection, and Score are from the AWA software for testing the circularity of a contig.

File name: Supplementary Data 3
Description: Present (green) and missing (gray) genes from the mitogenomes of 24 species of parasitic lice. Duplicate genes are indicated with numbers.

File name: Supplementary Data 4
Description: Number of gene arrangement differences among 11 individuals of lice in the taxon Columbicola passerinae 2 and two outgroup species (C. passerinae 1 and C. columbae).

File name: Supplementary Data 5
Description: Presence/absence matrix of gene arrangement differences among 11 individuals of lice in the taxon Columbicola passerinae 2 and two outgroup species (C. passerinae 1 and C. columbae). “M” indicates “Missing.”

File name: Supplementary Data 6
Description: Proportions of specific substitutions estimated from fourfold degenerate sites of lice from Columbicola (wing) and Physconelloides (body).
