# Supplementary material for: Independent evolution of highly variable, fragmented mitogenomes of parasitic lice
Source: Commun Biol. 2022 Jul 8;5:677. doi: 10.1038/s42003-022-03625-0 (PMC9270496; doi:10.1038/s42003-022-03625-0)
Supplement: Supplementary file 9 — Reporting Summary [file 42003_2022_3625_MOESM9_ESM.pdf]

nature portfolio

Corresponding author(s): Andrew Sweet

Last updated by author(s): May 26, 2022

Reporting Summary

Nature Portfolio wishes to improve the reproducibility of the work that we publish. This form provides structure for consistency and transparency in reporting. For further information on Nature Portfolio policies, see our Editorial Policies and the Editorial Policy Checklist.

Statistics

For all statistical analyses, confirm that the following items are present in the figure legend, table legend, main text, or Methods section.

n/a	Confirmed	
☐	☒	The exact sample size (*n*) for each experimental group/condition, given as a discrete number and unit of measurement
☒	☐	A statement on whether measurements were taken from distinct samples or whether the same sample was measured repeatedly
☐	☒	The statistical test(s) used AND whether they are one- or two-sided<br>*Only common tests should be described solely by name; describe more complex techniques in the Methods section.*
☒	☐	A description of all covariates tested
☒	☐	A description of any assumptions or corrections, such as tests of normality and adjustment for multiple comparisons
☐	☒	A full description of the statistical parameters including central tendency (e.g. means) or other basic estimates (e.g. regression coefficient) AND variation (e.g. standard deviation) or associated estimates of uncertainty (e.g. confidence intervals)
☐	☒	For null hypothesis testing, the test statistic (e.g. *F*, *t*, *r*) with confidence intervals, effect sizes, degrees of freedom and *P* value noted<br>*Give P values as exact values whenever suitable.*
☒	☐	For Bayesian analysis, information on the choice of priors and Markov chain Monte Carlo settings
☒	☐	For hierarchical and complex designs, identification of the appropriate level for tests and full reporting of outcomes
☒	☐	Estimates of effect sizes (e.g. Cohen's *d*, Pearson's *r*), indicating how they were calculated

Our web collection on statistics for biologists contains articles on many of the points above.

Software and code

Policy information about availability of computer code

Data collection	No software
Data analysis	Trimmomatic v.0.36, FastQC v.0.11.7, AWA, MITOS2, APE v.5.4, GEIGER v.2.0.7, aTRAM v.2.3.1, ABySS v.2.2, ade4 v. 1.7, Bowtie2 v.2.3.5.1, Geneious Prime v.2020.1.2 , RNAfold, GGPUBR v. 0.40, MAFFT v.7, MegaX v.10.1.8, RELAX, IQTree v.2.1.1, ModelFinder, PHYTOOLS v.0.7, CAPER v.1.0.1

For manuscripts utilizing custom algorithms or software that are central to the research but not yet described in published literature, software must be made available to editors and reviewers. We strongly encourage code deposition in a community repository (e.g. GitHub). See the Nature Portfolio guidelines for submitting code & software for further information.

Data

Policy information about availability of data

All manuscripts must include a data availability statement. This statement should provide the following information, where applicable:

- Accession codes, unique identifiers, or web links for publicly available datasets
- A description of any restrictions on data availability
- For clinical datasets or third party data, please ensure that the statement adheres to our policy

Data associated with this study are available in the Supplementary material, NCBI GenBank (novel sequences pending), NCBI SRA, or on the Dryad Digital Data Repository (doi.org/10.5061/dryad.9w0vt4bhx). Code used for generating results for this study are available on GitHub (https://github.com/adsweet/mitogenomes).

March 2021

Human research participants

Policy information about studies involving human research participants and Sex and Gender in Research.

Reporting on sex and gender	NA
Population characteristics	NA
Recruitment	NA
Ethics oversight	NA

Note that full information on the approval of the study protocol must also be provided in the manuscript.

Field-specific reporting

Please select the one below that is the best fit for your research. If you are not sure, read the appropriate sections before making your selection.

☐ Life sciences ☐ Behavioural & social sciences ☒ Ecological, evolutionary & environmental sciences

For a reference copy of the document with all sections, see nature.com/documents/nr-reporting-summary-flat.pdf

Ecological, evolutionary & environmental sciences study design

All studies must disclose on these points even when the disclosure is negative.

Study description	This study investigated the evolutionary patterns of mitochondrial genome structure in parasitic lice (Insecta: Psocodea: Phthiraptera). The authors used existing genomic sequence data to assemble mitochondrial genomes (mitogenomes) from a broad sample of parasitic lice, including representatives from every parvorder and most families. From these data, the authors used an existing phylogeny to reconstruct the evolutionary history of mitogenome fragmentation in lice, specifically testing how many times fragmentation arose in the group. The data were also used to compare nucleotide composition, instances of heteroplasmy, order of mitochondrial genes, and signals of relaxed selection associated with transitions from single to fragmented mitogenomes.
Research sample	Newly-assembled mitogenomes from 24 species of lice, combined with 49 existing mitogenomes (from NCBI GenBank); previously published mitogenome fragments from 11 individuals of a single species of louse (Columbicola passerinae)
Sampling strategy	The sampling strategy was chosen to maximize the mitogenomes from a taxonomically diverse sample of parasitic lice. This included data to match the taxonomic sampling from a recent phylogeny of parasitic lice and using all available mitogenomes of parasitic lice from NCBI GenBank.
Data collection	Obtained genomic data and assembled mitogenomes from NCBI SRA and GenBank databases; assembled mitogenomes using established computational pipelines and software; data collected by A.D. Sweet, K.P. Johnson, and S.L. Cameron.
Timing and spatial scale	Start: July 2018; Completion: December 2020.
Data exclusions	NA
Reproducibility	All methods are detailed in the Methods section of the manuscript. All data and code are available on NCBI SRA, NCBI GenBank, Dryad, GitHub, and the Supplementary Data files.
Randomization	NA
Blinding	NA

Did the study involve field work? ☐ Yes ☒ No

Reporting for specific materials, systems and methods

We require information from authors about some types of materials, experimental systems and methods used in many studies. Here, indicate whether each material, system or method listed is relevant to your study. If you are not sure if a list item applies to your research, read the appropriate section before selecting a response.

Materials & experimental systems

n/a	Involved in the study
☒	☐ Antibodies
☒	☐ Eukaryotic cell lines
☒	☐ Palaeontology and archaeology
☒	☐ Animals and other organisms
☒	☐ Clinical data
☒	☐ Dual use research of concern

Methods

n/a	Involved in the study
☒	☐ ChIP-seq
☒	☐ Flow cytometry
☒	☐ MRI-based neuroimaging
